# Supplementary material for: Activation of OSM-STAT3 Epigenetically Regulates Tumor-Promoting Transcriptional Programs in Cervical Cancer
Source: Cancers (Basel). 2022 Dec 10;14(24):6090. doi: 10.3390/cancers14246090 (PMC9775986; doi:10.3390/cancers14246090)
Supplement: Supplementary file 1 [file cancers-14-06090-s001.zip › cancers-2064359-supplementary.pdf]

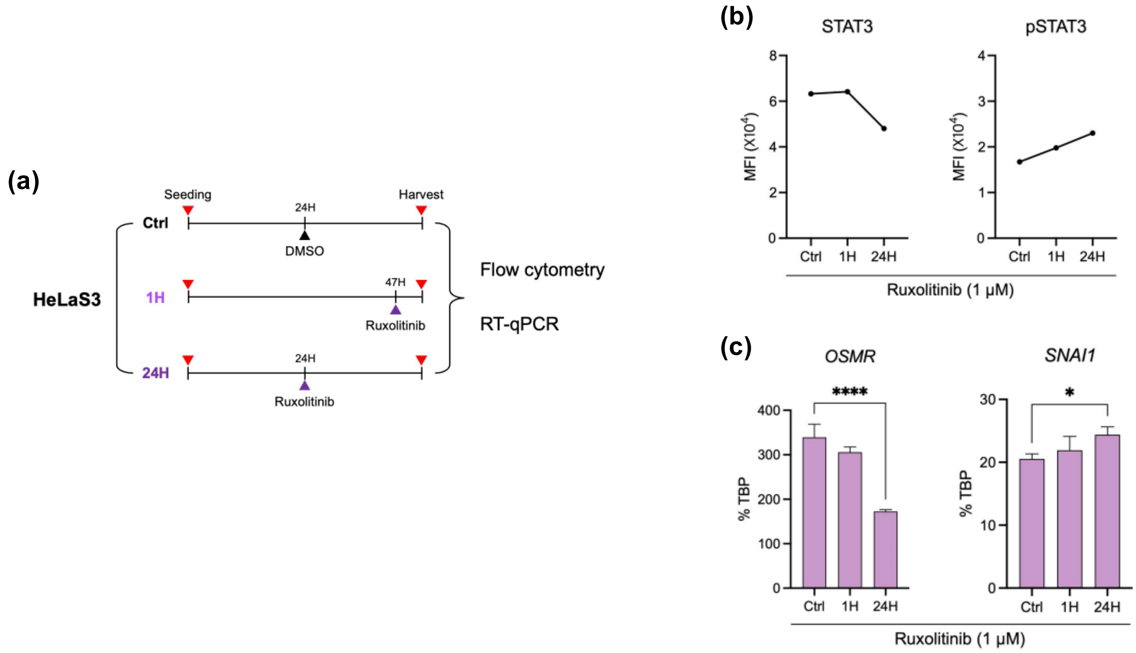

Figure S1. Regulation of STAT3 activity by ruxolitinib in cervical cancer cell. (a) Experimental design: HeLaS3 were exposed to Ruxolitinib (1 mM/ml) for 1 hour or 24 hours. (b) Analysis of STAT3, and pSTAT3 protein expression by flow cytometry in HeLaS3 during the 1H or 24H of Ruxolitinib stimulation as indicated. (c) Relative expression was normalized to internal control (TBP) and expressed relative to untreated, Ruxolitinib 1H, and 24H treated HeLaS3; *$p < 0.05$, ****$p < 0.0001$; Ordinary one-way ANOVA.
